# Advanced glycation end products (AGEs) and other adducts in aging-related diseases and alcohol-mediated tissue injury

**DOI:** 10.1038/s12276-021-00561-7

**Published:** 2021-02-10

**Authors:** Wiramon Rungratanawanich, Ying Qu, Xin Wang, Musthafa Mohamed Essa, Byoung-Joon Song

**Affiliations:** 1 Section of Molecular Pharmacology and Toxicology, Laboratory of Membrane Biochemistry and Biophysics, National Institute on Alcohol Abuse and Alcoholism, 9000 Rockville Pike, Bethesda, MD 20892 USA; 2 Neuroapoptosis Drug Discovery Laboratory, Department of Neurosurgery, Brigham and Women’s Hospital, Harvard Medical School, 60 Fenwood Road, Boston, MA 02115 USA; 3 Department of Food Science and Nutrition, Aging and Dementia Research Group, College of Agricultural and Marine Sciences, Sultan Qaboos University, Al-Khoud, Muscat, Oman; 4 Aging and Dementia Research Group, Sultan Qaboos University, Muscat, Oman

**Keywords:** Medical research, Experimental models of disease

## Abstract

Advanced glycation end products (AGEs) are potentially harmful and heterogeneous molecules derived from nonenzymatic glycation. The pathological implications of AGEs are ascribed to their ability to promote oxidative stress, inflammation, and apoptosis. Recent studies in basic and translational research have revealed the contributing roles of AGEs in the development and progression of various aging-related pathological conditions, such as diabetes, cardiovascular complications, gut microbiome-associated illnesses, liver or neurodegenerative diseases, and cancer. Excessive chronic and/or acute binge consumption of alcohol (ethanol), a widely consumed addictive substance, is known to cause more than 200 diseases, including alcohol use disorder (addiction), alcoholic liver disease, and brain damage. However, despite the considerable amount of research in this area, the underlying molecular mechanisms by which alcohol abuse causes cellular toxicity and organ damage remain to be further characterized. In this review, we first briefly describe the properties of AGEs: their formation, accumulation, and receptor interactions. We then focus on the causative functions of AGEs that impact various aging-related diseases. We also highlight the biological connection of AGE–alcohol–adduct formations to alcohol-mediated tissue injury. Finally, we describe the potential translational research opportunities for treatment of various AGE- and/or alcohol-related adduct-associated disorders according to the mechanistic insights presented.

## Metabolism: AGEs in aging- and alcohol-related diseases

Advanced glycation end products (AGEs), molecules formed when proteins and lipids combine with sugar, play key roles in aging-related diseases and in alcohol-induced tissue damage. AGEs naturally accumulate in the body with aging, and are also present in food, particularly in fried and processed foods. They are also produced by alcohol metabolism and cigarette smoking. Byoung-Joon Song and Wiramon Rungratanawanich at the National Institute on Alcohol Abuse and Alcoholism in Bethesda, USA, have reviewed how AGEs accumulate and their roles in aging-related diseases and alcohol-related health effects. They report that AGEs are linked to promotion or exacerbation of many diseases such as cardiovascular and kidney disease, adult-onset diabetes, cancer, and neurodegenerative conditions like Alzheimer’s and Parkinson’s diseases. The authors highlight opportunities for further research and for reducing AGE accumulation through changes in diet and behavior.

## Introduction

Advanced glycation end products (AGEs) are potentially harmful heterogeneous molecules of irreversible products derived from nonenzymatic glycation. Reactions involving nonenzymatic glycation occur between the reactive carbonyl group of a reducing sugar and nucleic acids, lipids, or proteins. AGEs can be formed endogenously or provided by exogenous sources under normal and pathological conditions^1,2^. The pathological implications of AGEs are ascribed to their ability to produce reactive oxygen (ROS) and nitrogen (RNS) species, as well as oxidative stress and inflammation, leading to structural and functional protein alterations, cellular dysfunction and apoptosis, and ultimately multitissue/organ injuries^3^. Cross-links formed by the interactions of AGEs with their cell surface receptors for advanced glycation end products (RAGEs) have been found during the development and progression of various aging-related diseases, such as diabetes, cardiovascular complications, kidney malfunctions, osteoporosis, cancer, neurodegenerative diseases, and liver disorders^4,5^

Alcohol (ethanol), one of the most addictive substances consumed by billions of people, has been found to cause more than 200 diseases and injuries worldwide^6,7^. In the United States alone, more than 250 billion dollars are lost annually due to alcoholism-related disorders and their consequences^8^. In addition, multiple millions of global deaths have been reported from excessive alcohol consumption due to acute and long-term consequences such as accidents, injuries, and a wide range of diseases^7,9^. Chronic misuse of alcohol promotes damage to multiple tissues and organs. It can lead to the development of various pathological states, such as alcohol use disorder, alcoholic liver disease, and alcohol-related brain damage, as well as increase the risk of cancer in the gastrointestinal tract, respiratory tract, breast, and liver^10,11^. However, despite the considerable amount of research in this area, the underlying molecular mechanisms by which alcohol exerts its cellular toxicity and organ damage remain to be further characterized.

In this review, we first briefly describe the properties of AGEs: their formation, accumulation, and RAGE interactions. We then focus on the causative functions of AGEs that impact various aging-related diseases. In addition, we highlight the biological connections of AGE–alcohol–adduct formations in alcohol-mediated multiorgan damage. Finally, we briefly describe the potential translational research opportunities for treating various AGE- and/or alcohol-related adduct-associated disorders based on the mechanistic insights presented.

## AGE formation

### Endogenous AGEs

Endogenous AGEs represent adducts that are produced and slowly accumulated within the body during the normal aging process and under the oxidative stress, inflammatory, and hyperglycemic (high blood sugar) conditions often observed in diabetes and other metabolic syndromes^1,4,12,13^. AGEs are formed by a nonenzymatic glycation reaction, also known as the Maillard reaction, between the carbonyl group of a reducing sugar and a free amino group (*N*-terminus, lysine, or arginine residue) of proteins or (adenine or guanine) of nucleic acids. This reaction is followed by a highly reversible nucleophilic addition reaction to generate a reversible Schiff base adduct, which is rearranged to a more stable and covalently bound Amadori product (e.g., hemoglobin A1c). Then, the Amadori product undergoes rearrangement, dehydration, and oxidation reactions to form irreversible products, AGEs, in the body. In addition to nonenzymatic glycation, AGEs can be formed through the polyol pathway and lipid peroxidation in the presence and absence of hyperglycemia, depending on the substrate type, reactant concentration, exposure time, and host cellular context^14–17^.

Various factors are involved in promoting AGE formation. These factors include excessive and/or prolonged alcohol consumption, cigarette smoking, the intake of high fat/caloric diets and/or extensively processed food, renal status, homeostatic imbalance, inflammation, hyperglycemia, and oxidative stress^2–4^. For example, persistently high blood glucose, often observed in people with type 2 diabetes, increases the reservoir of substrate for accelerating AGE formation and activates protein kinase C and NADPH oxidases, producing ROS^13,18^. Additionally, elevated oxidative stress can act as a catalyst to stimulate AGE accumulation, while activation of AGEs can increase oxidative stress, creating a synergistic feed-forward loop to accelerate pathophysiological conditions^2–4^.

### Exogenous AGEs

Exogenous AGEs include dietary AGEs (e.g., foods and beverages such as soft drinks containing high fructose corn sirup and/or sugar) and cigarette smoke. Additionally, dietary AGEs can be produced during food preparation. This formation of dietary AGEs depends on various factors, such as (1) temperature. e.g., the food browning process; (2) water content. e.g., dry-heat cooking; (3) pH status, e.g., food processing at high pH; and (4) cooking time and method, e.g., long-term cooking or storage and frying or broiling. These variations activate the nonenzymatic Maillard reaction, leading to the formation of glycation products and AGEs^5,19^. For example, high heat and prolonged cooking time enhance the rate and amount of AGE production in diets^20^. Foods with high pH values (up to 10) have elevated AGEs due to free amino groups under alkaline conditions^21^. Putative examples of dietary AGEs are modern Western diets such as bakery goods, breakfast cereals, cheese, and meat cooked by a dry-heat method^22^. In addition to dietary AGEs, cigarette smoke is found to contain reactive glycation products, which can increase AGE accumulation in the tissues and circulating blood of smokers^23^.

The amounts of AGEs in exogenous sources are usually much higher than those of endogenously produced AGEs^2–4,24^. Thus, it is likely that the intake of foods and/or beverages with high levels of exogenous AGEs create more health problems than endogenously produced AGEs, although both exogenous and endogenous AGEs are similar in their biological functions and potentially act synergistically to stimulate oxidative stress, inflammation, and cellular damage, contributing to detrimental pathophysiology^2–4,24^.

## AGE accumulation

### Intracellular AGEs

AGEs gradually accumulate during the aging process through normal metabolic and glycation activities. The aggregation of AGEs from endogenous and/or exogenous sources can negatively affect the functions of many cells in the entire body, resulting in diverse cellular responses and ultimately cellular damage and degeneration. Intracellular AGE accumulation can stimulate aberrant protein glycation, abnormal protein folding, and the aggregation of irregular or oligomeric proteins, as well as elevated oxidative stress and inflammation and upregulated apoptotic signaling pathways. These changes can contribute to protein inactivation, endoplasmic reticulum (ER) stress, mitochondrial dysfunction, cell apoptosis, and organ damage^25,26^. For instance, in neuronal tissues, the accumulation of AGEs induces glycation of α-synuclein and tau proteins, leading to protein dysfunction accompanied by the aggregation of harmful protein oligomers capable of initiating and developing neurodegenerative diseases^27,28^.

### Extracellular AGEs

AGEs are long-lived irreversibly formed molecules found in the circulatory system and tissues, particularly those with long-lasting proteins such as lens crystallins, cartilage, glomerular basement membrane, and extracellular matrix^29,30^. Cross-linking AGEs with extracellular matrix proteins, including laminin, elastin, and collagen, can alter the elasticity and function of tissues. In fact, higher levels of AGE cross-links are commonly detected in experimental animal models and autopsied tissue samples from people who are aging or have cancer, obesity, or diabetic complications^31–33^. In addition to long-lived proteins, AGEs can bind to proteins with short half-lives such as serum albumin, thereby activating RAGEs and inducing inflammatory responses followed by protein dysfunction and cell damage^34^.

## RAGEs and other AGE receptors

A RAGE is a multiligand cell surface receptor in the immunoglobulin superfamily^35^. Generally, RAGEs are expressed widely in various cells, such as endothelial cells, macrophages/monocytes, neutrophils, lymphocytes, microglia, astrocytes, and neurons, and organs, such as the brain, heart, lung, kidney, and liver^36,37^. However, under pathological conditions, RAGEs can be upregulated and participate in various aging-related pathophysiologies, such as adult-onset type 2 diabetes mellitus (DM), cardiovascular disorders, myocardial infarction, chronic kidney failure, pancreatitis, cancer, Alzheimer’s disease (AD), Parkinson’s disease (PD), hepatic fibrosis, and alcohol-mediated tissue injury^37,38^.

The interaction of AGE-RAGE triggers intracellular and extracellular signaling pathways through the activation of extracellular signal-regulated kinases 1 and 2 (ERK1/2), mitogen-activated protein kinases (MAPK), phosphoinositide 3-kinase (PI3K), protein kinase B (AKT), NADPH oxidase, and nuclear factor-κB (NF-κB). Activation of these proteins usually increases oxidative stress and inflammation that, in turn, promotes RAGE expression in a positive feed-forward loop, contributing to chronic disease development^39–41^. In addition to an AGE, a RAGE binds diverse ligands, such as members of the S100 protein family, amyloid-β peptides (Aβ), prions, and high-mobility group protein B1 (HMGB1), which alter cellular functions and contribute to various pathophysiologies^42–44^.

In addition to RAGEs, AGEs can interact with other cell surface receptors that possess the opposite function of RAGEs. These AGE cell surface receptors include AGE-R1, AGE-R2, AGE-R3, and scavenger receptors such as macrophage scavenger receptors, scavenger receptor class B type I and II (SR-BI, SR-BII), and cluster of differentiation 36 (CD36). The interaction of AGEs with these receptors can enhance their catabolism and clearance by modulating endocytosis and degradation^45,46^, suggesting a potentially adaptive defensive mechanism in the body to reduce the detrimental effects of increased glycation products.

## AGEs and aging-related diseases (see summary in Table 1)

### AGEs and diabetes

Chronic hyperglycemia, frequently observed in experimental models and human diabetes, exhibits elevated AGE formation, serum AGE levels, RAGE expression, and AGE-RAGE interactions. Consequently, these changes lead to increased oxidative stress, insulin resistance, inflammation, pancreatic β-cell dysfunction with apoptosis, and eventually diabetic complications, including retinopathy, neuropathy, cardiomyopathy, microvascular complications, and nephropathy^47–50^.

**Table 1:** Summary of the AGEs and AGE-RAGE interaction in aging-related diseases.

Diseases	Mechanisms	Consequences
Diabetes	↓ SIRT1, active PGC1α ↑ ROS, MAPK (JNK), NADPH oxidase	↑ oxidative stress, inflammation, mitochondrial dysfunction ↑ pancreatic β-cell dysfunction and apoptosis, insulin resistance, glucose -induced insulin secretion impairment= diabetic complications^51–55^
• Diabetic microvascular complications	↑ extracellular matrix glycation	↓ vascular elasticity ↑ vascular inflammation and permeability and blood-tissue barrier breakdown ↑ pericyte apoptosis^56,57^
Cardiovascular diseases	AGEs ≡ mononuclear, endothelial, and smooth muscle cells RAGE ≡ HMGB1 and S100 ↑ MAPK	↑ oxidative stress and inflammation ↑ oxidation of LDLs ↑ cardiomyocyte dysfunction, apoptosis, and tissue damage = severity of coronary atherosclerosis and coronary artery disease^55–64^
Kidney diseases	RAGE ≡ HMGB1 ↑ extracellular matrix glycation ↑ proximal tubular senesces ↑ NF-κB, MAPK, PKC, ERK1/2, MCP-1, TNF-α, IL-6, CTGF, TGF-β	↑ oxidative stress and inflammation ↑ mesangial cell proliferation inhibition, hypertrophy, and apoptosis ↑ podocyte damage, glomerular hypertrophy, and proteinuria ↑ fibrosis = renal failure and end-stage renal disease^65–75^
Obesity	RAGE ≡ HMGB1 and S100 ↑ HMGB1 and S100 ↑ JNK, IKK, NF-κB, TNF-α ↑ disruption of the hypothalamic function	↑ oxidative stress and inflammation ↑ body weight and energy intake ↑ hypothalamic insulin and leptin resistance = hypothalamic dysfunction^76–78^
Osteoporosis	RAGE ≡ HMGB1 and S100 ↑ ROS, NF-κB, MAPK, ERK1/2, TNF-α, IL-1β, caspase-3, Wnt, PI3K, VEGF, MMP-13	↓ mass and structural composition of bone ↓ osteoblast growth, differentiation, and apoptosis ↑ osteoclast generation^79–85^
Cancer	RAGE ≡ HMGB1 and S100 ↑ extracellular matrix glycation ↑ NF-κB, NADPH oxidase, VEGF, local hypoxia ↑ tumor-associated macrophages	↑ oxidative stress and inflammation ↑ epithelial-mesenchymal cell transition and migration ↑ cancer microenvironment as well as tumoral angiogenesis and proliferation ↑ cancer initiation, progression, migration, invasion, and metastasis^31,32,86–94^
Gut microbiome-associated diseases	↑ particular microbiome growth ↑ modulation of the composition and amounts of intestinal microflora ↑ proinflammatory cytokines, toxic metabolites, and bacterial products	↑ loss of microbial diversity ↑ intestinal epithelial cell damage, gut barrier dysfunction, intestinal permeability, and bacterial translocation = systemic endotoxemia, inflammation and multiorgan injury^95–101^
Neurodegenerative diseases	↑ reactive gliosis ↑ NF-κB	↑ oxidative stress and cellular stress ↑ activated gliosis = neuronal death and degeneration^4,102–108^
• Alzheimer’s disease	RAGE ≡ Aβ and HMGB1, AGE- albumin ↓ SIRT1 ↑ Aβ, tau, and amyloid precursor protein (APP) ↑ phosphorylated tau ↑ cross-linked AGE-Aβ and their aggregates ↑ AGE-albumin adducts ↑ NF-κB, JNK, ERK1/2, caspase-3, PI3K, Bax, iNOS, p38, JAK/STAT	↑ oxidative stress ↑ Aβ aggregation ↑ activated gliosis = neuronal apoptosis and neurodegeneration^109–113^
• Parkinson’s disease	AGEs ≡ α-synuclein RAGE ≡ S100 ↑ NF-κB and TNF-α	↑ aggregation of α-synuclein toxic oligomers ↑ Lewy body formation ↑ activated gliosis ↑ death of the dopaminergic neurons = neurodegeneration^114–119^
Liver diseases	↓ GSH, SIRT1, and TIMP3 ↑ ROS, NF-κB, NADPH oxidase, MAPK ↑ phosphorylation of IRS-1, JNK, c-JUN, IKK, UCP-2, ERK1/2	↑ oxidative stress and inflammation ↑ inflammatory cell death of parenchymal cells and tissue remodeling process fibrosis/cirrhosis ↑ cell apoptosis, hepatocyte dysfunction, and steatosis = induce initiation and progression of NAFLD = Inflammatory liver injury [nonalcoholic steatohepatitis (NASH)], hepatic fibrosis and cirrhosis^55–57,120–131^

↑ increase/activate, ↓ decrease/inactivate, = lead to/result in, ≡ interact/cross-link.

Dietary AGEs have been found to disrupt and downregulate sirtuin1 (SIRT1) expression, resulting in the acetylation and inactivation of peroxisome proliferator-activated receptor γ coactivator-1α (PGC1α), a master regulator of mitochondrial metabolism. AGEs also stimulate mitochondrial ROS production and c-Jun N-terminal protein kinase (JNK) and NADPH oxidase activity to induce mitochondrial dysfunction and oxidative stress^51–53^. Furthermore, increased oxidative stress and JNK/MAPK expression can contribute to pancreatic β-cell dysfunction with apoptosis, glucose-induced insulin secretion impairment, and subsequent insulin resistance, which is a key hallmark of type 2 DM^54^. Restriction of dietary AGEs can reduce and/or reverse these effects by enhancing insulin sensitivity and upregulating AGE-R1 and SIRT1 expression while suppressing NF-κB, tumor necrosis factor-alpha (TNF-α), leptin, and serum AGEs in type 2 DM^55^.

AGEs play important roles in diabetic microvascular complications by cross-linking with extracellular matrix proteins, thereby altering vascular elasticity, structure, and function^56^. In addition, the AGE-RAGE interaction further instigates pericyte apoptosis, vascular inflammation and permeability, and blood-tissue barrier breakdown^56^. In contrast, the inhibition of RAGE by RAGE antiserum can prevent the toxicity induced by AGEs in diabetic microvascular complications^57^.

### AGEs and cardiovascular diseases

AGEs can stimulate cardiovascular complications in the presence or absence of hyperglycemia^58^. The accumulation and exposure to AGEs exacerbate oxidative stress and inflammation and initiate the oxidation of low-density lipoproteins (LDLs), which are harmful to cardiovascular function. In blood vessels, accumulated AGEs also interact with mononuclear, endothelial, and smooth muscle cells, resulting in cellular dysfunction, tissue damage, and atherosclerosis development^55–57,59^. In acute myocardial infarction, increased expression of RAGE and its interaction with AGEs, HMGB1, and S100 induce cardiomyocyte apoptosis by activating the MAPK pathway^60,61^. Additionally, higher levels of circulating AGEs are positively correlated with the incidence of cardiovascular disorders and severity of coronary atherosclerosis and coronary artery disease independent of diabetic status^62–64^, suggesting a causal role of AGEs in cardiovascular diseases.

### AGEs and kidney diseases

The kidney is a highly specialized organ that reabsorbs many essential molecules, including water and salt, while removing potentially toxic compounds to protect the body. The accumulation of AGEs in the kidney can elevate oxidative stress and inflammation, contributing to renal failure^65^. In humans, serum contains AGE peptides and free AGE adducts. AGE peptides are generally filtered by renal glomeruli and further reabsorbed by proximal convoluted tubules and eliminated from the body in the form of free AGE adducts in the urine^66,67^. Increased circulating AGEs can accumulate in the renal glomeruli and enhance collagen and laminin production in the extracellular matrix together with proximal tubular senescence, oxidative stress, and inflammatory processes^66^. On the other hand, acute/chronic kidney disease and end-stage renal failure can reduce AGE clearance. Consequently, glomerular and tubular cells are exposed to potentially harmful AGEs for extended periods of time owing to the lower rates of glomerular filtration. These changes lead to accelerated progression and/or exacerbation of kidney malfunction and nephropathy along with greater amounts of AGEs in the circulation^68,69^.

In addition, AGEs can be produced in renal mesangial cells and induce the expression of monocyte chemoattractant protein-1 (MCP-1). AGEs also activate NF-κB, MAPK, and protein kinase C (PKC) to promote mesangial proliferative inhibition, hypertrophy, and apoptosis^70,71^. Similarly, the AGE-RAGE interaction in the kidney can increase oxidative stress, inflammation, and fibrosis by stimulating connective tissue growth factor (CTGF), transforming growth factor-β (TGF-β), MAPK, NF-κB and PKC pathways to develop podocyte damage, glomerular hypertrophy, proteinuria, and ultimately end-stage renal failure^66,72,73^. The RAGE ligand HMGB1 also plays a causal role in renal inflammation by enhancing ERK1/2, TNF-α, interleukin (IL)-6, and MCP-1, leading to nephropathy and chronic kidney disease^74,75^.

### AGEs and obesity

Dietary and endogenous AGEs and the AGE-RAGE interaction can promote oxidative stress and inflammation, contributing to the accelerated progression of obesity-related complications such as elevated serum AGEs, insulin resistance, AGE accumulation, and elevated proinflammatory cytokines in adipose tissues^67,76^, although consumption of dietary AGEs does not necessarily increase obesity based on its marginal association with increased body weight gain^67,76^. Adipose tissues also produce molecular RAGE ligands (such as HMGB-1) and RAGE-inducible molecules (e.g., MCP-1 and IL-6). Upon RAGE interaction, these bound molecules activate their own production in adipose tissues, suggesting a causal role of RAGE signaling in the inflammatory pathway^49,67^. In addition to dietary AGEs, endogenous AGEs can be trapped and accumulated in adipose tissues, and AGE accumulation can be prevented by RAGE inhibition^77^.

AGEs are reported to modify energy balance by disrupting hypothalamic function. Aggregated AGEs can activate the JNK, Iκ-B kinase (IKK), NF-κB, and TNF-α pathways to trigger hypothalamic insulin and leptin resistance, resulting in hypothalamic dysfunction, imbalanced energy control, and subsequently elevated food consumption and body weight with obesity and metabolic syndromes^78^.

### AGEs and osteoporosis

Body bone mass is determined by the delicate balance between osteoclasts and osteoblasts involved in regulating bone formation, differentiation, and apoptosis in response to various stimuli^79^. In bone, accumulated AGEs can increase osteoclast generation while decreasing osteoblast growth and differentiation. Initially, AGEs enhance the levels of osterix, a transcription factor that promotes osteoblast differentiation and bone formation. However, chronic AGE accumulation induces osteoblast apoptosis through activation of proapoptotic caspase-3, MAPK, and intracellular ROS generation^80–82^, indicating an intricate role for AGEs in bone physiology and remodeling.

The interaction of AGE-RAGE augments the production of proinflammatory cytokines and suppresses osteoblast differentiation via the Wnt, PI3K, and ERK1/2 signaling pathways^83^. Moreover, RAGE binding to its ligands (such as HMGB1 and S100) also triggers the production or activation of TNF-α, IL-1β, caspase-3, MAPK, NF-κB, vascular endothelial growth factor (VEGF), and matrix metalloproteinase 13 (MMP-13), negatively affecting the mass and structural composition of bone^84^. Notably, several conditions, including aging, diabetes, renal failure, tobacco smoking, and excessive alcohol consumption that induce AGE accumulation and AGE-RAGE interaction, are risk factors for increased bone fracture and osteoporosis^46,85^.

### AGEs and cancer

Elevated amounts of AGEs are observed in tumor tissues in cancer. Increased AGEs and AGE-RAGE interactions provide a link to cancer initiation, progression, migration, and metastasis^31,86^. The binding of AGEs to RAGE triggers extracellular matrix glycation, NADPH oxidase activity, local hypoxia, VEGF expression, and NF-κB activation to produce oxidative stress and inflammation, supporting the cancer microenvironment and promoting tumoral angiogenesis and proliferation^32,87^.

The overexpression of RAGE and its interaction with a ligand are associated with various types of cancer. RAGE is highly upregulated in metastatic and aggressive breast, ovarian, and pancreatic cancer, acting as a promotor of the progression of premalignant precursors to invasive carcinoma^88,89^. In hepatocellular carcinoma (HCC), RAGE overexpression and interaction with HMGB1 induce tumor-associated macrophage activation and NF-κB expression to promote tumoral proliferation, invasion, and metastasis^90,91^. After binding with S100, RAGE also contributes to the epithelial-mesenchymal cell transition and cell migration, which is associated with tumor invasion and metastasis in cervical cancer and osteosarcomas, suggesting a contributing role for RAGE in tumor malignancy^92^. Furthermore, in cigarette smokers, elevated levels of RAGE are positively correlated with the development of oral squamous cell, lung, and breast carcinoma^93,94^.

### AGEs and gut microbiome-associated diseases

The gut microbiota plays a critical role in regulating body function by producing diverse metabolites and influencing the gut-liver-brain axis and other pathways, such as immune system pathways. The composition and function of gut flora can be affected by endogenous and exogenous factors, particularly food consumption with different levels of dietary AGEs^95,96^.

In the body, less than 30% of dietary AGEs are absorbed in the intestine after ingestion, and less than 15% are excreted in urine and feces, leading to a hypothesis that the remaining unabsorbed AGEs are degraded by gut microorganisms^97^. The intestinal microbiota produces deglycating enzymes to digest AGEs, which are utilized for energy production. As a result of this mechanism, unabsorbed AGEs may play a role in modulating the composition and number of intestinal microflora^98^.

AGEs can modify gut microbiota composition by triggering the growth of particular microbiomes, resulting in the loss of microbial diversity and an increased possibility of intestinal leakiness^98,99^. Elevated accumulation of AGEs can enhance gut barrier dysfunction, intestinal permeability, and bacterial translocation by stimulating the production and release of proinflammatory cytokines, potentially toxic metabolites and bacterial products, as well as causing intestinal epithelial cell damage, contributing to systemic endotoxemia, inflammation, and multiorgan injury^98^. AGE-modulated gut microflora is also associated with the pathogenesis of type 2 DM, obesity, neurodegenerative diseases such as AD, and end-stage renal failure, where restriction of dietary AGEs can improve the gut microbial composition and subsequently attenuate disease conditions^100,101^.

### AGEs and neurodegenerative diseases

The brain is a highly specialized organ with tightly regulated motor, behavior, neurocognitive, and executive functions. However, it generally lacks defensive or protective enzymes/proteins compared to peripheral tissues such as the liver and kidney^102^. Thus, under normal conditions, the brain is protected by a special functional system, the blood-brain barrier (BBB). In the brain, AGEs can be produced as a result of elevated oxidative stress during aging or chronic exposure to toxic agents such as alcohol (ethanol), n-6 fatty acid-containing high-fat Western diets, and sugary soft drinks. Oxidative stress can also be generated due to AGE formation in the brain. This feed-forward process creates a vicious cycle that exacerbates oxidative damage with the subsequent initiation and progression of neurodegenerative diseases^4,103^. However, the amounts of plasma AGEs, which can stimulate the aggregation of specifically modified proteins, are different in Alzheimer’s and Parkinson’s disease patients in a sex-dependent manner, thus requiring a careful interpretation of test results^104^. Similar to AGEs, RAGEs are significantly or markedly expressed in many areas of the brain, such as the cortex, hippocampus, cerebellum, and substantia nigra^105–107^ during the development of neurodegenerative diseases. Activated microglial cells produce and secrete AGE-albumin to induce RAGE expression in neurons and promote neuronal cell death, thereby contributing to neurodegenerative disorders^4,108^. Additionally, the accumulation of AGEs and the AGE-RAGE interaction greatly induce reactive gliosis and the NF-κB proinflammatory pathway, leading to cellular stress, activated gliosis, and eventually neuronal degeneration^105^.

#### AGEs and Alzheimer’s disease

Various forms of AGEs are markedly aggregated in neuronal plaques in the brain as well as in the serum and cerebrospinal fluid (CSF) in experimental models and autopsied brains from AD patients compared with the corresponding controls. Accumulated AGEs can accelerate the formation of Aβ, tau, and amyloid precursor protein (APP) and induce the hyperphosphorylation of tau and the cross-linking of AGE-Aβ, leading to an increase in these aggregates^109^. AGEs also suppress SIRT1 expression and stimulate inducible nitric oxide synthase (iNOS) and caspase-3 to enhance neuronal apoptosis and/or degeneration with elevated gliosis^110^.

One of the most abundant AGE-protein adducts in the brain is the AGE-albumin adduct, which was confirmed by mass spectral analysis, and causes RAGE overexpression in primary neurons in human AD brains. The formation of the AGE-albumin adduct is intensified by elevated oxidative stress and Aβ aggregation^111^. Aβ is also produced by the AGE-albumin-RAGE interaction, which in turn supports AGE-albumin adduct formation in a positive feed-forward cycle. The binding of the AGE-albumin adduct to RAGE provides a link to neuronal apoptosis by activating the proapoptotic JNK and Bcl-2-associated X protein (Bax) pathways^111^. In addition to AGEs and AGE adducts, HMGB1 and Aβ can bind RAGEs to activate the NF-κB, ERK1/2, p38, JNK, PI3K, Janus kinase/signal transducers and activators of transcription (JAK/STAT) pathways, leading to neuronal cell death and neurodegeneration^112^. Furthermore, AGEs cross-linked with Aβ can also decrease the ability of microglia to clear plaques^113^.

#### AGEs and Parkinson’s disease

Dietary AGEs promote AGE formation in the substantia nigra^114^. In PD brains, AGEs can accumulate early in newly formed Lewy bodies, suggesting that AGEs could play a contributing role in Lewy body formation in developing PD^115^. AGEs cross-linked with α-synuclein are also present in PD brains, resulting in the aggregation of α-synuclein toxic oligomers^115,116^. Moreover, RAGE can interact with S100 in PD brains, activating the NF-κB and TNF-α signaling pathways to promote dopaminergic neuronal death and subsequent neurodegeneration in PD^117,118^. AGE-albumin, the most abundant AGE product in the human PD brain, is synthesized by activated microglial cells. Aggregated AGE-albumin upregulates RAGE, leading to the apoptosis of primary dopamine neurons in the brain^119^.

### AGEs and liver diseases

The liver plays a vital role in the metabolism and synthesis of various essential molecules and proteins needed for many other organs. It is involved in the catabolism and elimination of circulating AGEs using liver sinusoidal endothelial cells and Kupffer cells. This function of the liver declines during the aging process and in various liver diseases, resulting in the accumulation of AGEs or their aggregates^120,121^.

Intracellular AGE accumulation is observed in animal models of hepatic steatosis and other advanced liver diseases, such as hepatic inflammation (steatohepatitis) and fibrosis/cirrhosis. Accumulated AGEs in hepatocytes can stimulate apoptosis and inflammation, leading to cellular dysfunction, steatosis, and ultimately nonalcoholic fatty liver disease (NAFLD)^121–123^.

Endogenous and exogenous AGEs provoke the initiation and progression of NAFLD. However, consumption of dietary AGEs from sources such as fructose- or sucrose-enriched diets and/or soft drinks can worsen liver fibrosis faster than consumption of endogenous AGEs. The aggregation of AGEs decreases the levels of the most important cellular antioxidant peptides, glutathione (GSH), SIRT1, and tissue inhibitor of metalloproteinase 3 (TIMP3), accompanied by increased oxidative stress and inflammation, promoting inflammatory liver injury and fibrosis in experimental animal models and NAFLD patients^55–57,124–126^.

Accumulated AGEs and AGE-RAGE interactions in these cells augment the generation of ROS and the activation of MAPK and NF-κB pathways, leading to inflammatory cell death in the parenchyma and tissue remodeling processes during fibrosis^127,128^.

Increased amounts of RAGE are also observed in the liver of hepatocellular carcinoma (HCC), and its level is significantly greater than it is liver affected by hepatitis and healthy liver specimens. The amounts of serum AGEs are also higher in HCC than in NASH and healthy liver specimens. In HCC, the AGE-RAGE interaction is associated with angiogenesis and tumor proliferation and invasion, which are ameliorated by RAGE inhibition. These results suggest an important role for AGEs and RAGE in promoting NAFLD, NASH, fibrosis/cirrhosis, and HCC pathogenesis^129–131^.

## AGEs and alcohol-mediated tissue injury

### Alcohol metabolism (see summary in Fig. 1)

#### Hepatic alcohol metabolism

The major type of alcohol that is consumed is ethanol (CH_3_CH_2_OH). Upon consumption, ethanol is absorbed via simple diffusion in the small intestine into the blood and rapidly circulates throughout the body^6^. Ethanol metabolism primarily occurs in the liver in three major steps: (1) ethanol oxidation to acetaldehyde, (2) acetaldehyde metabolism to acetate, and (3) acetate catabolism to H_2_O and CO_2_^132–134^.

**Fig. 1:**
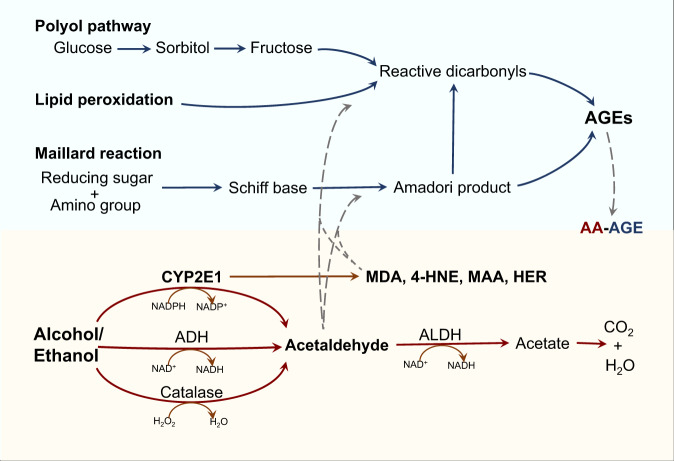
Overview of the biological connections in AGE–alcohol–adduct formations. Under conditions that increase lipid peroxidation, polyol pathway, and Maillard reaction, such as exposure to alcohol and/or high n-6 fat diets or high fructose drinks, different AGEs are produced. Ethanol and its reactive metabolites generated by CYP2E1 are also likely involved in the AGE synthesis pathways to produce the final acetaldehyde and AGE (AA-AGE) adducts. The formation of AA-AGE adducts can be observed after chronic alcohol exposure. These AA-AGE adducts exhibit similar properties (e.g., brown color and polymerization) as the AGE adducts cross-link with sugar molecules, and they are different from MAA adducts formed by AA and MDA interactions. However, treatment with an antioxidant can halt AA-AGE adduct formation, supporting the idea that AA-AGE adducts can be generated from a Schiff base product similar to AA adducts and AGEs.

In step I of the hepatic oxidative metabolism of alcohol (ethanol), three distinct enzymes are involved: alcohol dehydrogenase (ADH), catalase, and cytochrome P450-2E1 (CYP2E1). Cytosolic ADH is a major enzyme that catalyzes the oxidative metabolism of ethanol to acetaldehyde by using the cofactor nicotinamide adenine dinucleotide (NAD^+^) converted to NADH^132–134^. Catalase present in peroxisomes may participate in ethanol oxidation in the presence of H_2_O_2_^132–134^, although this enzyme appears to play no major role in hepatic ethanol metabolism under physiological conditions due to the limited supply of H_2_O_2_^135^. After chronic and/or large amounts of ethanol intake, CYP2E1, the major component of the microsomal ethanol oxidizing system (MEOS) with a higher km (~10 mM for ethanol) than that of ADH (km <1 mM for ethanol), is also involved in ethanol metabolism through an NADPH-dependent pathway. In contrast to those of ADH and catalase, CYP2E1 expression and activity are usually induced by ethanol and other substances, such as dietary fats. The induction and activation of CYP2E1, in turn, contribute to alcohol- and nonalcohol-induced pathophysiology^136–144^. In the second step, acetaldehyde is converted to acetate due to the low km (for acetaldehyde) mitochondrial aldehyde dehydrogenase 2 isozyme (ALDH2) in humans^145^. Notably, CYP2E1 also takes part in this step with NADPH acting as a cofactor to convert acetaldehyde to acetate^146^. Finally, the acetate that is produced is further degraded to CO_2_ and H_2_O, ending the final step of ethanol oxidation in the liver^132–134^. Furthermore, the remaining unmetabolized ethanol, acetaldehyde, and acetate can be distributed to many other organs, including the heart, lung, kidney, pancreas, and brain, causing damage in various peripheral tissues and neurobehavioral effects caused by damage to the brain.

#### Brain alcohol metabolism

Similar to the liver, ethanol oxidizing enzymes are also functional in the brain and include ADH, catalase, CYP2E1, and ALDH2, which make different contributions. Cerebral ADH plays very little or virtually no role in ethanol metabolism in the brain compared to its action by its hepatic isoform. However, cerebral catalase acts as the main oxidizing enzyme, accounting for more than 60% of ethanol oxidation in the brain under normal conditions^147^. However, catalase may have a limited role in ethanol metabolism in certain brain regions, except for aminergic neurons, where it is present in high concentrations^134,148–150^. In contrast, CYP2E1 is widely expressed throughout the brain, such as in the cerebral cortex, hippocampus, and cerebellum. Similar to that in the liver, brain CYP2E1 is also inducible by ethanol and may therefore play a key role in cerebral ethanol metabolism, especially after chronic and/or binge alcohol intake^151–155^. The role of CYP2E1 in brain ethanol metabolism and its association with alcohol-mediated oxidative neuronal injury^155–160^ and aging-related AD and PD^161^ have also been suggested. Finally, mitochondrial ALDH2 is the final enzyme for converting acetaldehyde to acetate^148^, although acetaldehyde is locally produced in the brain due to its very limited ability to cross the BBB^162^. In contrast, ALDH1B and other isoforms may be involved in acetaldehyde metabolism in rodents^145,147,163^.

### AGE–alcohol–adduct formations (Fig. 1)

Free amino acid residues of proteins, lipids, nucleic acids, and nucleophilic molecules are major targets of adduct formation or covalent binding with reactive molecules such as acetaldehyde, acrolein, crotonaldehyde, formaldehyde, malondialdehyde, 4-hydroxynonenal, 8-hydroxydeoxyguanosine, N2-((furan-2-yl)methyl)-2′-deoxyguanosine, and N^2^-ethyl-2′-deoxyguanosine^164–166^. After alcohol consumption, CYP2E1 in step I of oxidative ethanol metabolism can generate a series of oxygen free radicals (e.g., ROS) to trigger lipid peroxidation, resulting in the formation of various ethanol metabolites and adducts, including (1) acetaldehyde (AA) from ethanol oxidation, (2) malondialdehyde (MDA) and (3) 4-hydroxynonenal (4-HNE) from lipid peroxidation, (4) malondialdehyde–acetaldehyde adduct (MAA) from the acetaldehyde-MDA-protein hybrid adduct, and (5) hydroxyethyl radicals (HER) from the presence of iron during ethanol metabolism^167–171^, although some of these adducts may not be readily detected due to their low levels under physiological conditions.

MDA and 4-HNE are reactive lipid peroxides with short half-lives that can form covalent adducts with various proteins and nucleic acids in the body. For instance, ethanol intake can induce 4-HNE to interact with cytochrome C oxidase (complex IV) and ALDH2 in the mitochondria, GRP78 and disulfide isomerase in the ER, and ERK1/2, phosphatase and tensin homolog (PTEN), AMP-activated protein kinase (AMPK), and gamma-glutamylcysteine synthetase (GCS) in the cytosol. These adduct formations inactivate their target proteins and result in the accumulation of lipid aldehydes, ER stress, mitochondrial dysfunction, cell signaling alteration, and potentially multitissue injury^172–177^.

Interactions between acetaldehyde or MDA and cellular proteins lead to the formation of MAA adducts, which are highly stable and resistant to rapid degradation^178,179^. Increased levels of MAA adducts from alcohol exposure stimulate protein dysfunction and immune-induced tissue injury through interactions with Toll-like receptor-3 (TLR-3), TLR-6, calpain, collagen alpha-1 (XII) chain, procollagen type XIV alpha-1, protocadherin beta, or complement component proteins^167,180^.

Acetaldehyde adducts (AA adducts) are formed by the interaction of acetaldehyde, a direct metabolite of ethanol oxidation and a human carcinogen, with certain amino acids, including lysine, cysteine, and aromatic amino acids. However, these amino acids in different proteins may exert an unequal preference for AA adduct formation. The proteins commonly bound to acetaldehyde to produce AA adducts include membrane proteins of the red blood cells (erythrocytes), hemoglobin (oxygen transport), tubulin (cellular structure), lipoproteins (lipid transport), albumin (blood), and collagen (connective tissue)^167,169,180^. In addition, some of these AA adducts are produced in a CYP2E1-dependent manner^137,181^.

The formation of adduct proteins can prolong protein half-lives and accumulate as aggregated proteins. Since mammalian ubiquitin-dependent proteasomal degradation is usually catalyzed after the conjugation of ubiquitin with lysine residues, it is expected that AA adduct formation and ubiquitin conjugation may compete for common lysine residues. Therefore, when lysine residues are already occupied by reactive acetaldehyde, lipid aldehydes, AGEs, or AA, adducts persist for extended periods of time and may end up as aggregated proteins due to the lack of free lysine needed for ubiquitin conjugation and subsequent proteolysis. In fact, the hypothesis of extended half-lives and aggregations of these adduct proteins is exemplified by the stabilization of CYP2E1 by ethanol and acetone through the blockade of its rapid degradation via ubiquitin-dependent proteolysis^182–186^.

AA adducts are produced in two separate pathways that yield two different types of adducts, depending on the existing conditions. The first pathway is the formation of AA adducts with specific amino acids (lysine, cysteine, or aromatic amino acids) and *N*-ethyl amino acid groups under reducing conditions. The second is AA adduct synthesis under nonreducing conditions, that creates a wide range of adducts for which the complete mechanisms remain to be further characterized. In the second pathway, the initial step is the formation of a Schiff base adduct followed by several rearrangements and reactions to yield diverse AA adducts^168^.

Under conditions that include increased lipid peroxidation, such as exposure to alcohol and/or high n-6 fat diets or intake of high fructose drinks, different AGEs are produced by Schiff base formation (Fig. 1). Thus, ethanol and its reactive metabolite, acetaldehyde, are likely involved in the AGE synthesis pathways to produce the final acetaldehyde and AGE (AA-AGE) adducts. The formation of AA-AGE adducts can be observed after chronic alcohol exposure. These AA-AGE adducts exhibit similar properties (e.g., brown color and polymerization) as the AGE adducts cross-link with sugar molecules, and they are distinguished from MAA adducts formed by AA and MDA interactions. Furthermore, treatment with an antioxidant can halt AA-AGE adduct formation, supporting the idea that AA-AGE adducts can be generated from a Schiff base product similar to AA adducts and AGEs^168^.

#### Brain (see summary in Table 2)

Chronic and excessive alcohol consumption can alter brain structure and function, causing behavioral, emotional, and intellectual abnormalities. These neurobehavioral changes include the development of alcohol tolerance and addiction, emotional dysregulation, and executive, neurocognitive, and motor dysfunctions with neuroinflammation and/or neurodegeneration. Excessive alcohol exposure increases oxidative stress and the levels of reactive acetaldehyde and lipid aldehydes with a simultaneous decrease in defensive molecules and detoxification enzymes, including GSH and ALDH2^53,172,177^. These changes subsequently lead to the accumulation of acetaldehyde adducts and possibly AGEs in the brain, which has a much lower detoxification capacity than the liver, as previously mentioned^102^. Additionally, reactive electrophilic acetaldehyde can strongly induce covalent adduct formation with nucleophilic molecules such as proteins and DNA, forming acetaldehyde-protein (AA-protein) or acetaldehyde-DNA (AA-DNA) adducts in the brain^187–189^.

**Table 2:** Summary of the biological connections of AGE–alcohol–adducts with alcohol-mediated tissue injury.

Cells/organs	AGE–alcohol–adducts	Consequences
Brain	AGE-albumin adducts	↑ RAGE overexpression ↑ MAPK (JNK and p38K), Bax, and microglial activation ↑ neuronal inflammation, apoptosis, and damage^108,111,119,202^
	Ethanol	↑ RAGE expression ↑ ROS, Nrf2, GFAP, Iba1, lipid peroxidation, HMGB1, TLR-4, neuroimmune markers, ↑ CYP2E1, oxidative stress, and inflammation ↑ neuroinflammation, neuronal apoptosis, and memory Impairment^203,204^
	AA-protein adducts: AA-tubulin adducts AA-DA adducts (salsolinol)	↓ microtubule formation ↑ dysfunction of cytoskeletal components of nerve cells ↑ neurotoxin involved in the pathogenesis of AUD and PD ↑ neuronal damage and degeneration^189–198^
	AA-DNA adducts	↓ DNA integrity and neuronal viability ↓ represses DNA repair enzymes/system ↑ genetic instability and DNA mutations ↑ correlated with AUD^53,192,197,198^
	AA-AGE adducts	↑ ROS and oxidative stress ↑ neurotoxicity, neuronal apoptosis, and degeneration in a dose-dependent manner^199^
Liver	AGE production plasma AGEs AGE-protein adducts	↓ decrease in albumin turnover in plasma ↑ aggregation of adduct proteins ↑ activation of Kupffer and HSCs ↑ death of hepatocytes^232,233^
	AA adducts MDA adducts MAA adducts 4-HNE adducts HER adducts Ethanol	↑ TNF-α, IL-12, IL-18, MIF, PDGF ↑ stimulate the transformation of the HSCs ↑ activate myofibroblasts ↑ activates infiltration of neutrophils into the liver↓ ALDH2 function^214–230^ ↑ CYP2E1, oxidative stress, AFLD, and advanced ALD^203–213^
	AA-AGE adducts	↑ RAGE expression ↑ ROS and oxidative stress ↑ hepatic fatty degeneration and steatosis ↑ hepatocyte ballooning, apoptosis, and steatosis ↑ ALD and AFLD^215,231^
Lung	soluble RAGE (+HMGB1)	↑ lung inflammation^239,240^
	MDA adducts 4-HNE adducts	↑ oxidative stress ↑ pulmonary dysfunction ↑ lung epithelial barrier dysfunction ↑ acute respiratory distress syndrome (ARDS)^164,234^
	MAA adducts	↓ impedes the wound healing process ↑ inflammatory processes, PKC-mediated release of IL-8 ↑ correlated with AUD^179,235–238^
Heart	MDA adducts 4-HNE adducts	↓ ALDH2 function ↑ oxidative stress ↑ cardiac dysfunction^241–244^
Gut	AA-adducts AA-MDA adducts	↑ oxidative stress ↑ leaky gut with increased intestinal cell permeability and endotoxemia^137,181,245–250^
Pancreas	4-HNE adducts HER adducts	↑ pancreatitis, β-cell apoptosis^251–255^
Erythrocytes	AA adducts	↑ correlated with FASD^257,258^
Testis	RAGE overexpression	↑ oxidative stress and inflammation↑ testis dysfunction and degeneration^253,259–261^

↑ increase/activate, ↓ decrease/inactivate.

AA-protein adducts are widely present in the brains of alcohol-exposed rodents or people with alcohol use disorder (AUD)^189–192^. After alcohol consumption, AA-protein adducts are rapidly produced in the cerebral cortex primarily with mitochondrial proteins and localized in the white matter, deep layers of the frontal cortex, and molecular layer of the cerebellum^188,189,191^. AA-protein adducts are also formed with cytosolic proteins, including tubulins, known as AA-tubulin adducts, resulting in microtubule malformation and the cytoskeletal dysfunction of nerve cells^193,194^. Furthermore, acetaldehyde can interact with dopamine (DA) to produce AA-DA adducts, including salsolinol, a neurotoxin and potent stimulant in alcohol, which is involved in the pathogenesis of AUD and PD^194,195^. Additionally, the aggregation of these AA-protein adducts can enhance neuronal damage and degeneration in different brain regions^190,196^. These findings suggest a critical role for AA-protein adducts in promoting brain damage and pathophysiology associated with chronic alcohol consumption.

The presence of acetaldehyde in the brain also alters DNA integrity and neuronal viability. Acetaldehyde can form AA-DNA adducts in the brain and in peripheral blood leukocytes of people with AUD since ethanol, acetaldehyde, and lipid aldehydes such as MDA and 4-HNE can inhibit DNA repair enzymes^53^. The dual mechanisms of simultaneously elevated AA-DNA adducts and suppressed DNA repair systems following alcohol intake may contribute to genetic instability and DNA mutations, promoting neurological pathologies^192,197,198^.

In addition to cerebral AA-protein adducts, ethanol intake or exposure can induce and accelerate AGE production in the brain. Aggregation of cross-linked AGEs further upregulates RAGE expression and activation, contributing to diverse outcomes with neurobehavioral impairment, as observed in people with AUD^190^.

In neurons, direct exposure of AA-AGE adducts to cortical neurons triggers ROS generation, leading to oxidative stress and consequently neuronal apoptosis and degeneration in a dose-dependent manner. These effects can be prevented by treatment with the antioxidant *N*-acetylcysteine (NAC) or a neutralizing anti-AA-AGE antibody, suggesting a direct role for AA-AGE adducts in generating oxidative stress-induced neurotoxicity^199^. Daily alcohol exposure for ten consecutive days also instigated microglial activation to synthesize and secrete AGE-albumin adducts in the hippocampus and entorhinal cortex of the rat brain. Accumulated AGE-albumin adducts increase the expression of RAGE and activate the MAPK (JNK and p38K)-dependent cell death pathway to promote inflammation, apoptosis, and neuronal damage, which are significantly attenuated by treatment with soluble RAGE and chemical AGE inhibitors^200^. Moreover, ethanol ingestion also upregulates RAGE expression in the orbitofrontal cortex and increases the levels of oxidative stress, HMGB1, TLR-4, neuroimmune markers, and proinflammatory cytokines in the brain^201^. Additionally, chronic exposure to alcohol enhances RAGE expression, ROS generation, nuclear factor erythroid 2–related factor 2 (Nrf2), TLR-4, glial fibrillary acidic protein (GFAP), ionized calcium-binding adapter molecule 1 (Iba1), and lipid peroxidation, resulting in neuroinflammation, neuronal apoptosis, and memory impairment^202^. These results strongly suggest a contributing role of the AGE-RAGE axis in alcohol-induced neuroinflammation and neurodegeneration through elevated oxidative stress and upregulated cell death pathways in the brain.

#### Liver

Chronic and excessive alcohol intake is known to cause liver injury from mild steatosis (simple fat accumulation in so-called alcoholic fatty liver disease, AFLD) to more advanced liver disease such as inflammation (alcoholic steatohepatitis, ASH), fibrosis/cirrhosis, hepatic cancer, liver failure, and death^203–213^. Although many aberrant signaling pathways are found in the pathogenesis of liver diseases, elevated levels of adducts with AA, MDA, MAA, 4-HNE, and HER likely contribute to alcohol-induced cell injury^214,215^. After ethanol intake, AA, MDA, and 4-HNE adducts are produced rapidly in the hepatic centrilobular zone (zone III), sinusoids, and HSCs as well as on the hepatocyte surface in the liver. The amounts of these adducts are also markedly elevated in the early phase of alcoholic liver disease (ALD) in the presence or absence of clinical or histological signs^216–219^, suggesting their role in the initial development and progression of ALD. In addition to individual AA and MDA adducts, MAA adducts (AA-MDA-protein hybrid adducts) are found in HSCs, liver sinusoidal endothelial cells, and Kupffer cells in ALD models. They also stimulate the transformation of HSCs into activated myofibroblasts, resulting in the elevated production of potent profibrotic factors such as platelet-derived growth factor (PDGF)^220–222^. The presence of MAA adducts in these cells can induce proinflammatory cytokines such as TNF-α, IL-12, IL-18, and macrophage migration inhibitory factor (MIF)^223–225^. In hepatic Kupffer cells, 4-HNE adducts are also present and activate the infiltration of neutrophils into the liver, leading to the activation of HSCs and an inflammatory cytokine response^226–228^. These findings indicate a contributing role of MAA and 4-HNE adducts in promoting the hepatic immune response associated with ALD development. In advanced ALD with cirrhosis, 4-HNE and MDA adducts are also found in greater amounts than in nonalcoholic liver cirrhosis and normal liver^139,228^, suggesting the involvement of 4-HNE and MDA adducts in promoting advanced ALD. Finally, adducts derived from HER are also detected in the liver and localized in the microsomes and plasma membranes of hepatocytes and are inducible by alcohol exposure^229,230^.

Ethanol consumption stimulates the formation of AA-AGE in the liver. Increased AA-AGE adduct formation and accumulation were positively correlated with the progression of ALD, as indicated by hepatic fat accumulation and steatohepatitis, which are reversibly ameliorated by alcohol abstinence^215^. HSCs directly exposed to AA-AGE adducts can enhance RAGE expression, ROS generation, and oxidative stress to promote hepatocyte ballooning, apoptosis, and steatosis^231^. These findings suggest a role for AA-AGE adducts in the pathogenesis of AFLD. Alcohol ingestion also enhances AGE synthesis and plasma AGEs in the circulation, especially in the portal and hepatic veins of cirrhotic livers. Elevated AGE levels support AGE-protein adduct formation accompanied by decreased albumin turnover in plasma^232^. Additionally, AGE-protein adducts can accumulate as aggregated proteins in the cells, leading to the activation of Kupffer and HSCs in the liver and microglial cells in the brain followed by parenchymal cell death (e.g., hepatocytes and neurons, respectively), suggesting the involvement of AGEs in the progression of ALD and alcohol-mediated multiorgan injury^233^.

#### Other organs

Although more detailed studies need to be conducted, the involvement of AGEs – alcohol –adducts in different cells/tissues is presented as follows:

##### Lung

Chronic and/or excessive alcohol intake can cause acute respiratory distress syndrome (ARDS) and other types of pulmonary dysfunction characterized by reduced air exchange rates with increased lung epithelial barrier dysfunction, possibly through increased oxidative stress and MDA and 4-HNE adducts^164,234^. Ethanol intake stimulates MAA adduct formation in the lung. Elevated levels of MAA adducts are found in the pulmonary bronchoalveolar lavage fluids obtained from chronically alcohol-exposed animals and in people with AUD^179,235^. MAA adducts bind scavenger receptor A (SR-A) on lung macrophages to activate inflammatory processes in airway epithelial cells^236^. The formation of MAA adducts also impedes the wound healing process of bronchial epithelial cells and activates PKC-mediated release of IL-8 in the lung. However, these changes are attenuated by treatment with PKC inhibitors^237,238^. In addition to adduct formation, ethanol exposure can increase soluble RAGE expression in bronchoalveolar lavage fluid together with RAGE ligand (HMGB1) in the inflammatory lung^239,240^.

##### Heart

Excessive alcohol consumption is known to cause cardiac dysfunction with reduced blood ejection force and volume, possibly by augmenting oxidative stress, increasing the levels of MDA and 4-HNE adducts and suppressing ALDH2 activity^241,242^. Elevated oxidative stress caused by alcohol oxidation increases acetaldehyde and MDA adduct synthesis in the heart^243,244^. However, the contributing roles of AA-MDA adducts in alcohol-mediated myocardiopathy need to be further characterized. Since CYP2E1 and ALDH2 are expressed in the heart, future studies of the opposite regulation of CYP2E1 (i.e., induction) and ALDH2 (i.e., suppression) during the alcohol-mediated production of MDA and/or 4-HNE adducts and the consequent cardiomyopathy would be of interest.

##### Gut

Excessive alcohol intake is known to cause leaky gut with increased intestinal cell permeability and endotoxemia, possibly via increased CYP2E1 and oxidative stress^245–248^. Ethanol ingestion is likely to increase AA formation in the intestine as a consequence of gut bacteria-mediated alcohol metabolism and the low levels of ALDH2 expression in the gut^249^, although additional studies are needed to extrapolate the roles of these factors in alcohol-mediated gut damage and inflammation. Since CYP2E1 was shown to be involved in the production of hepatic AA- and AA-MDA adducts^137,181,250^, it would be of interest to study the direct role of CYP2E1 in alcohol-mediated AGE production and GI pathologies.

##### Pancreas

Consumption of alcohol enhances the expression of 4-HNE and HER adducts as well as inflammation in the pancreas^251,252^. The functional role of these adducts may be related to alcohol-mediated pancreatitis, although this area needs to be evaluated in the future. Since CYP2E1 and ALDH2 are also expressed in the pancreas^253–255^, future study of the opposite regulation of CYP2E1 (i.e., induction) and ALDH2 (i.e., inactivation) during the production of MDA and/or 4-HNE adducts and the subsequent damage may be important for understanding the pathogenesis mechanisms of alcohol-mediated pancreatitis and dysfunction.

##### Serum

Ethanol intake is known to increase the amounts of MDA adducts and AGEs in the serum. The amounts of MDA adducts are more positively correlated with AGE concentrations than with blood glucose levels^256^. However, the effect of elevated MDA adducts in serum on alcohol-related multiorgan damage needs to be investigated in the future.

##### Erythrocytes

After alcohol consumption, AA adducts are produced in blood erythrocytes and are also correlated with the incidence of fetal alcohol spectrum disorders (FASDs)^257,258^. The pathological mechanisms and implications of these findings need to be evaluated in future studies.

##### Testis

Excessive alcohol consumption is known to cause testis dysfunction and degeneration, possibly by increasing oxidative stress. Alcohol intake can induce RAGE overexpression in the testis, whereby RAGE is localized in the interstitial cells and the basal compartment of the seminiferous tubules. Prolonged ethanol exposure stimulates RAGE activation to produce oxidative stress and inflammatory mediators, leading to testicular dysfunction and degeneration^259^. CYP2E1 and ALDH2 are also expressed in the testis^253,260,261^. Thus, it would be of interest to find the opposite regulations (i.e., induction of CYP2E1 with suppression of ALDH2) and their implications in alcohol-mediated production of MDA and/or 4-HNE adducts and subsequent testis dysfunction.

## Translational applications

We have thus far described the formation of various AGE adducts, interactions with many cellular components, including RAGE, and their pathological implications in aging-related diseases and alcohol-mediated multiorgan damage. Considering the underlying molecular mechanisms of these processes and functional implications, we can suggest a variety of different strategies for use in the effective prevention and therapy of AGE-associated tissue injury and/or RAGE-related diseases. For instance, we can reduce the amounts of potentially harmful AGEs by decreasing the production of endogenous AGEs to prevent many disease states, including aging-related diseases, and by lowering the dietary intake of exogenous AGEs. In fact, decreased intake of n-6 fatty acids in high-fat Western diets, dairy products, and soft drinks with high fructose or sucrose contents in conjunction with increased consumption of n-3 fatty acid-enriched fish and plant-based foods such as legumes, vegetables, fruits, and whole grains are recommended to decrease AGE levels in the body. These healthy dietary choices are consistent with the food recommendations of the American Diabetes Association, the American Heart Association, and the American Institute for Cancer Research^262–264^. Many herbs and spices, such as cloves, rosemary, and curcumin, and natural antioxidants, such rice (*Oryza sativa* L.), and blueberry, exhibit antiglycation activities. These foods are rich in polyphenols such as gallic acid, flavonoids, anthocyanin, and ferulic acid, which attenuate protein glycation and prevent the biosynthesis of AGEs^265–270^. As mentioned above, cooking methods can play a critical role in regulating the levels of AGE formation, with effects ranging from those caused by oven-frying > frying > broiling > roasting > boiling/poaching/stewing/steaming. For example, cooking meat (e.g., chicken, pork, or beef) by boiling or stewing can reduce the AGE contents to one-half of that prepared by broiling^1,271^. In addition, the water content, cooking method, temperature and time, and food pH are crucial to the final amount of AGEs. Marinating food or meat with acidic ingredients such as lemon juice and vinegar can decrease the amounts of dietary AGEs produced during the high-heat cooking process by as much as 50%^272^. These culinary methods are commonly used for traditional Asian, Mediterranean, and other cuisines worldwide to create palatable and healthy dishes. In addition, changes in behavior or lifestyle, such as decreasing the amount and frequency of alcohol intake and tobacco smoking, and engaging in physical exercise to reduce obesity and diabetes can lower the production of endogenous AGEs, subsequently preventing AGE-associated disease conditions.

We have also described the roles of increased oxidative stress in promoting the production of AA, MDA, MAA, and other AGE-related adducts and the consequences in aging-related diseases and alcohol-mediated multiorgan damage. In particular, CYP2E1 contributes to the production of AA and MAA adducts, as demonstrated in experimental rodent and cell culture models^137,180,216,273^. Therefore, the inhibition of oxidative stress-producing enzymes such as CYP2E1^137,181^ and NADPH oxidase^274^ is an option to prevent the formation of AGE adducts and AGE-associated cellular and/or organ damage. For instance, taking naturally occurring CYP2E1 inhibitors (e.g., diallyl disulfide in garlic^273,275^; phenyl isothiocyanate in cabbage and cruciferous vegetables^276^; ellagic acid in pomegranate^247^; polyunsaturated fatty acids, including docosahexaenoic acid (22:6n-3)^277^ and indole-3-carbinol in vegetables and fruits^278^; berberine in fruits and vegetables^279^; walnut^280^; curcumin^281,282^; quercetin^283^; and synthetic compounds (chlormethiazole and YH-439^137,284^) can prevent alcohol-induced oxidative stress and the formation of various adducts, including AA adducts^137^ and AA-MAA adducts^53,285^, although the preventive effects of all these dietary compounds on AGE-associated adduct formation have not been specifically evaluated. In addition, dietary AGEs decreased the amounts of SIRT1^131^ and other defensive proteins, including peroxisome proliferator -activated receptor-γ coactivator 1-α (PGC-1α)^51–53^ and ALDH2^172^. Subchronic alcohol intake also decreased the levels of SIRT1 and PGC-1α and other isoforms^286,287^. Thus, activation of SIRT1 by resveratrol and its synthetic structural derivatives^288,289^ and melatonin^290^ may prevent adduct formation and AGE-associated disease conditions. Furthermore, consumption of anti-inflammatory antioxidants from natural dietary supplements^279,281,282,290^ and/or synthetic origins^199^ can help reduce the incidence and severity of AGE-associated inflammation and related disorders. Furthermore, soluble RAGE^119,200^, inhibitors of the RAGE signaling pathway^130,131^, neutralizing antibodies against RAGE^57^ or AA-AGE adduct^199^, and other AGE-degrading compound(s), such as pyridoxamine and ALT-711^200^, lipoic acid^291^, synthetic compounds OPB-9195^292^, and nitrothiadiazolo[3,2-α]pyrimidines^293^, have been reported to decrease AGE adduct formation. Based on these findings, lifestyle changes that include decreased alcohol consumption, reduced tobacco smoking, and avoidance of potentially harmful diets along with increased physical exercise and daily intake of vegetables and fruits, should be actively promoted and undertaken for proper management of the levels of AGE-related adducts to help prevent aging-related diseases and alcohol-mediated organ damage.

## Concluding remarks (see overview in Fig. 2)

AGEs can be produced endogenously and exogenously. The accumulation of AGEs and the interaction of AGE-RAGE play causative roles in various aging-related diseases and alcohol-mediated tissue injuries by interfering with cell signaling pathways and forming adducts with cellular macromolecules, contributing to their inactivation and pathophysiology in many tissues/organs. These suggest biological connections between AGEs and alcohol adduct formation in relation to alcohol-mediated inflammation, gut leakiness, and multiorgan damage. In addition, the information described in this review can be useful to understand the underlying mechanisms of various diseases caused by alcohol intake, nonalcoholic substances, environmental risk factors, genetic mutations, and aging. These mechanistic insights can serve as a springboard for future translational applications for both the prevention and treatment of diverse disease states.

**Fig. 2:**
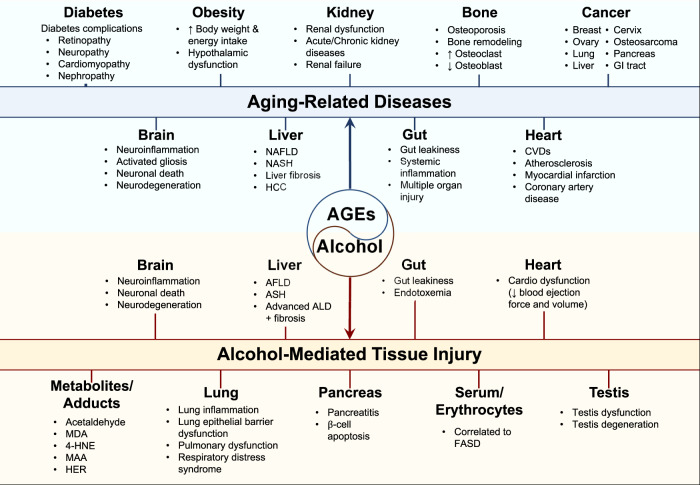
Overview of the causative functions of AGEs in aging-related diseases and alcohol-mediated multiorgan dysfunction or damage. AGEs can be produced endogenously and exogenously. The accumulation of AGEs and the interaction of AGE-RAGE play causative roles in various aging-related diseases and alcohol-mediated tissue injuries by interfering with cell signaling pathways and forming adducts with cellular macromolecules, contributing to their inactivation and pathophysiology in many tissues/organs. These suggest biological connections between AGEs and alcohol adduct formation in relation to alcohol-mediated inflammation, gut leakiness, and multiorgan damage.
